# Knowledge, Attitudes and Practice of Diabetes in Rural Bangladesh: The Bangladesh Population Based Diabetes and Eye Study (BPDES)

**DOI:** 10.1371/journal.pone.0110368

**Published:** 2014-10-14

**Authors:** Fakir M. Amirul Islam, Rahul Chakrabarti, Mohamed Dirani, M. Tauhidul Islam, Gail Ormsby, Mohamed Wahab, Christine Critchley, Robert P. Finger

**Affiliations:** 1 Department of Statistics, Data Science and Epidemiology, Faculty of Health, Arts and Design, Swinburne University of Technology, Melbourne, Australia; 2 Organisation for Rural Community Development, Dariapur, Narail, Bangladesh; 3 Centre for Eye Research Australia, Royal Victorian Eye and Ear Hospital, University of Melbourne, Melbourne, Australia; 4 Department of Physics, University of Melbourne, Melbourne, Australia; 5 Avondale College of Higher Education, Cooranbong, Sydney, Australia; 6 Narail Diabetes Hospital and Narail Diabetes Shamity, Narail, Bangladesh; University of Louisville, United States of America

## Abstract

**Background:**

To assess the Knowledge, Attitudes and Practice (KAP) amongst the general community regarding type 2 diabetes mellitus (DM) in rural Bangladesh.

**Methods:**

Data was collected using cluster random sampling from 3104 adults residing in a rural district in Bangladesh. Participants underwent a KAP questionnaire survey regarding assessing diabetes, socio-demographic and medical history. Descriptive, Chi-square and regression analyses were performed.

**Results:**

Participants were aged between 30 and 89 years (M  = 51, SD  = 11.8) and 65.5% were female. The prevalence of diabetes was found to be 8.3%. The majority (93%) reported to have heard of diabetes, yet only 4% knew what a glucose tolerance test was. Only 50% reported that they knew physical inactivity was a risk factor. Age, gender, level of education and socio-economic status (SES) were significantly associated with KAP. A lower proportion (41%) of older participants (aged ≥65 years) reported that they knew that dietary modifications assist in diabetes control compared to those aged less than 35 years (69%), p<0.001. Males (β  = 0.393, 95% CI = 0.142–0.643), and any level of education compared to no schooling (β  = 0.726, 95% CI = 0.596, 0.857) reported significantly more knowledge, after multivariate adjustments for covariates. Participants aged under 35 years, (odds ratio (OR)  = 1.73, 95% CI  = 1.22–2.43) had significantly higher positive attitudes towards treatments of diabetes compared to those aged ≥65 years. Of the 99 people with known diabetes, more than 50% (n = 52) never had their blood sugar levels checked since diagnosis.

**Conclusions:**

Knowledge of diabetes and its risk factors is very limited in rural Bangladesh, even in persons diagnosed with type 2 DM. The development of public health programmes to increase knowledge of diabetes and its complications is required to assist people living in rural Bangladesh to control and management of diabetes.

## Introduction

Globally, 70% of type 2 diabetes occurs in low resource countries [1]. Bangladesh is an example of a low-resource country, where the current prevalence of diabetes is estimated to be 6.3%. This may be underreported given the fact that the prevalence is higher in some other South East Asian Countries, for example 9.1% in India, 14.8% in Mauritius [1]. The surge in diabetes in low-resource settings is partly attributed to the insidious nature of the condition, with many people remaining undiagnosed until complications such as vision loss and renal disease manifest. Consequently, large proportions of people remain undiagnosed or fall within pre-diabetes categories which predisposes them to progressing to diabetes [1]. Several studies have shown that in low-resource countries a range of social determinants including poor health literacy are critical in the epidemiologic transition of disease outcome [2]–[4]. A growing body of evidence from knowledge, attitude and practice (KAP) studies have supported the need for greater awareness of prevention, diagnosis, risk factor control and disease management [2]–[7]. For example, KAP studies from South India have shown that individuals who are educated and diligent with their diabetes mellitus (DM) self-care gain longer term control [3]. Most studies of KAP related to diabetes have focussed on people with diagnosed DM or newly diagnosed DM attending urban clinics or hospitals [2], [3], [6]. However, there is a paucity of evidence assessing the implications of KAP regarding diabetes and its complications amongst the general community specifically in rural and remote areas.

In the last decade, a number of studies have reported the prevalence and risk factors of diabetes both in urban and rural areas of Bangladesh [8]–[12]. However, no study has reported KAP regarding diabetes, its risk factors or management in a general population before starting any cross-sectional or population-based studies. One exception was in 2012 when Saleh et al conducted a KAP survey amongst a sample of 508 patients with newly diagnosed type 2 diabetes attending outpatient departments of healthcare centres in urban Bangladesh [13]. The authors reported that 84% of respondents had at best an “average” basic knowledge of diabetes (using the University of Michigan Diabetes Knowledge Test), and 90% of respondents did not test their blood glucose regularly [13]. Similarly, a study conducted in the general population in a low resource Asian country Mongolia in 2010 reported that 20% of the participants had never heard the term diabetes prior to surveying, and one-third were unaware that the disease could be prevented through lifestyle changes [2]. Complementing this was randomised control trial evidence from Australia which showed that knowledge of the risk factors of diabetes and motivation to life style change were powerful predictors of change in diet and exercise, and associated with a significant reduction in body mass index, waist circumference and fasting blood glucose [14], [15]. However, the challenge remains in developing countries.

In this study, we aim to determine the level of KAP of diabetes, its risk factors and the variation according to the socio-demographic factors in a rural setting of Bangladesh.

## Materials and Methods

### Study population

Bangladesh is a country of over 148 million people divided into 64 districts. Each district is divided into Upazilas (sub-districts), and each Upazila is divided into Unions which consist of villages. The study location Banshgram is a union of Narail district located approximately 200 km southwest of the capital city Dhaka and has an eligible population within the age range of approximately 5,500 [16].

The sample consists of 3104 participants (65%, n = 2032 female) aged 30–89 years recruited using cluster random sampling from each of the total 18 villages from the Banshgram Union. The sample size was based on the prevalence of diabetes in adults in Bangladesh of 6.3% in 2012 estimated by the International Diabetes Federation’s Diabetes Atlas [1]. The sample size 3104 was sufficiently large enough to detect a minimum 5% difference in the proportion of attaining knowledge or attitudes of DM related items between males and females, no schooling and primary or secondary level of education (statistical power >90%, p = 0.05).

### Recruitment Strategy

The recruitment strategy involved identifying participants aged ≥30 years from each of the selected households within 18 clusters of villages. Recruitment started from the far east corner of a village and continued until at least 50% of the total eligible adults were interviewed from each of the villages. Participants were required to attend for two days to complete data collection. The first day involved an interview where information on KAP of diabetes and other socio-demographic factors including level of educational attainment and socio-economic status (SES) was collected and on day two they were subjected to a clinical examination to collect fasting capillary glucose and other anthropometric measurements.

### KAP Questionnaire

A questionnaire was developed to collect data on participant’s KAP regarding diabetes, its risk factors and management. In addition, data relevant to socio-demographic characteristics, and lifestyle factors and medication use for diabetes and hypertension were included in the questionnaire. The questions relevant to KAP in the questionnaire were derived from the validated instruments: (1) Knowledge and Awareness of Diabetes Questionnaire developed for the Chennai Urban Rural Epidemiology Study [17], (2) AusDiab Health Knowledge, Attitudes and Practices Questionnaire 99/00 [18], and (3) KAP construction guides [19]. Three tools were used to develop our questionnaire because we used the tool that was used in India by Mohan et al. [17] considering the fact that the people in Bangladesh and India are expected to follow a similar life style patterns. However, a question regarding quitting smoking was sourced from the AusDiab [18] measure. KAP construction guides [19] was used only as a guide to conduct our KAP survey but none of the questions were adopted from the guide. Questions evaluating knowledge of diabetes were associated with the categorical responses such as “yes” or “no” and were:

1. Have you ever heard of diabetes?
2. Do you know what is glucose tolerance test?

If you have heard of diabetes:

3. Do you know how to measure diabetes?
4. Do you know diabetes causes eye disease?
5. Do you know diabetes is due to genetics/hereditary?
6. Do you know diabetes can be controlled by regular exercise?
7. Do you know diabetes can be controlled by avoiding carbohydrate?
8. Do you know diabetes can be controlled by avoiding sugar?
9. Do you know diabetes can be controlled by avoiding smoking?

Question evaluating attitudes towards treatment of diabetes was associated with the categorical response “yes”, “no”, or “don’t know” by asking: When you or your family member or friend has diabetes, should they seek treatment? The attitude was considered to be positive towards treatment of diabetes if they had a positive response. Questions evaluated practice of diabetes control and management particularly for those who have been diagnosed with diabetes by asking:

1. Are you on medication?
2. How often do you have your blood sugar checked?

Frequently (more than once a year) = 1
Regularly (at least once a year) = 2
Whenever I consider my diabetes has become worsen = 3
Never after diagnosis = 0

The socio-demographic data included gender, age in categories (35, 35–44, 45–54, 55–64, ≥65 years), educational level in categories (no schooling, primary school 1–5 years, high school 6–10 years, School Secondary Certificate (SSC) or above) and socio-economic status (SES). The recruitment of study population, recruitment strategy, KAP questionnaire and socio-demographic factors are reported in detail elsewhere [20].

### Ethics approval

The research was conducted in accordance with the tenets of the Declaration of Helsinki and was approved by Human Research Ethics Committee for the Bangladesh Medical Research Council (Reference: BMRC/NREC/2010–2013/68). Written consent was obtained from the participants who were able to sign and verbal consent was obtained from those who were unable (47%) prior to inclusion. In the case of verbal consent, the data collector signed the consent form for the participants with their approval and the ethics committee approved this consent procedure. Participants were informed of their rights to withdraw from the study at any stage or to restrict their data from the analysis.

### Statistical analysis

Participant’s socio-demographic characteristics including age, gender, level of education and SES were reported using descriptive statistics. The items related to the knowledge of diabetes were compared between gender, age categories, level of education and SES using Chi-square tests. The Linear-by-Linear association option in Chi-square test was used to present p values for ordinal trends for all categorical variables. Rasch analysis, a form of item response theory (IRT) that transforms ordinal or binary categories into interval-level estimates [21], [22] was performed to compute person measures based on the nine knowledge items in a logarithmic scale and termed as “knowledge score” here after. Linear regression was applied to estimate the β coefficient for the outcome variable “knowledge score” for each of the socio-demographic characteristics adjusting for age, gender, education level, SES, diabetes status and religion. The negative value of a knowledge score indicates the person has below average knowledge, zero value indicates average and positive value indicates above average level of knowledge of the items. The interaction of gender with age and education; age with education; and education with religion and SES were also investigated and reported graphically for those were found to be statistically significant. The interactions were investigated due to the fact that the proportion of younger women was higher than men, women are less educated than men, younger people are more educated than older people, lower education attainment is associated with low SES. Logistic regression techniques were used to report odds ratio (OR) and 95% confidence interval (CI) for positive attitudes towards treatment of diabetes. Practice of diabetes for those with known diabetes were reported using simple descriptive statistics. Statistical software SPSS (SPSS Inc, version 21) and Winstep software (version 3.75.0) for Rasch analysis were used for analytical purpose.

## Results

Of the total 3104 participants 65.5% (n = 2032) were females, 47.1% (n = 1462) did not have any formal education, and 13.6% (n = 423) had insufficient funds most or all of the time in the previous year before data collection. In the age group of those aged older or equal to 65 years, males had a higher recruitment rate (i.e., 20%) compared to females (12%); and males had a higher level of education (13% had School Secondary Certificate (SSC) or above) compared to females (4% had SSC or above) (Table 1).

**Table 1:** Socio-Demographic Characteristics of the study participants of the Bangladesh Population based Diabetes and Eye Study (BPDES) (N = 3104).

		Number	%
Age, years	Less than 35 years	240	8
	35–44	876	28
	45–54	942	30
	55–64	590	19
	Above or equal to 65	456	15
Gender	Female	2032	65
	Male	1072	35
Religion	Muslim	2599	84
	Hindu	505	16
Level of Education	No education	1462	47
	1–5 primary	921	30
	6–10 High school	495	16
	SSC or above	226	7
Socio-economiccondition	Insufficient funds all or most the time	423	14
	Insufficient funds some of the time	1077	35
	Balance	1320	43
	Sufficient funds most of the time	268	9

### Knowledge Assessment

**Figure 1:**
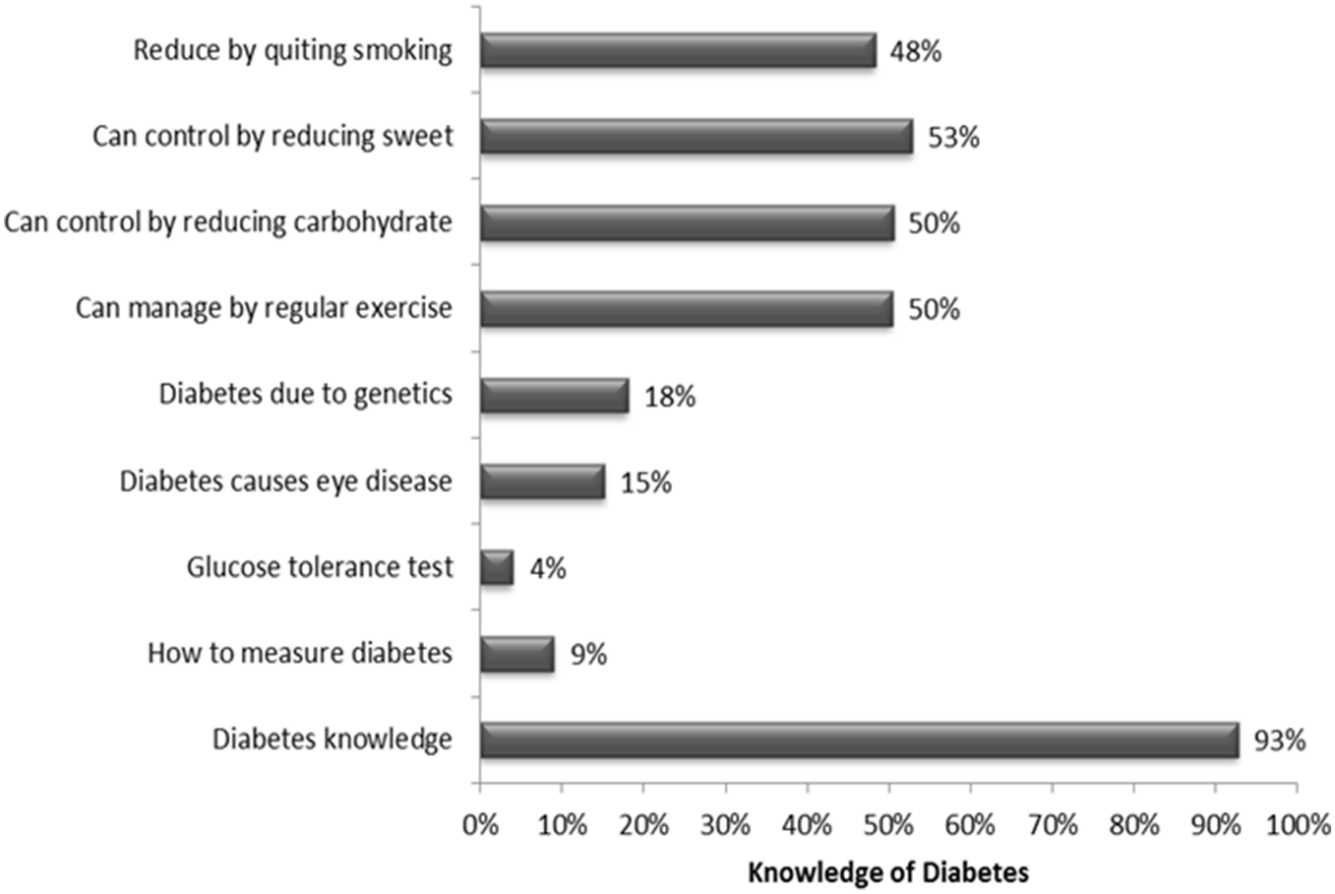
General Knowledge of Diabetes, its risk factors and management (N = 3104).

There was wide variation in the knowledge items, 4% to 93% (Figure 1). Overall, males had significantly better knowledge compared to females. That is, males were more likely than females to know that diabetes caused eye disease (18% vs. 13%), and diabetes could be controlled by regular exercise (55% vs. 48%). People in older age groups showed significantly poorer knowledge in relation to six of the items, compared to the people in younger age groups. For example, a significantly lower proportion (39%) of older participants (65 years or older) reported that they knew that regular physical activity can prevent diabetes compared to those aged less than 35 years (60%), p<0.001. Both people with at least SSC level of education and/or people with higher SES showed better knowledge on all of the items, for example, a significantly higher proportion (38%) of people with at least SSC level of education reported that they knew diabetes caused eye diseases compared to those with no schooling (9%) p<0.001 (Table 2).

**Table 2:** General Knowledge (known or heard about) of Diabetes and Common Eye Disease by Gender, Religion, Age, Education and Socio-economic Condition (N = 3104).

	Gender					Religion					Age groups*				
	Female(N = 2032)		Male(N = 1072)			Muslim(N = 2599)		Hindu(N = 505)			Age, <35(n = 240)		Age, > = 65(N = 456)		
	n	%	N	%	P	n	%	n	%	P	N	%	n	%	P for trend
Diabetes	1880	93	991	92	0.83	2452	94	419	83	<0.001	231	97	402	88	<0.001
Glucose tolerancetest (GTT)	68	3	54	5	0.02	99	4	23	5	0.42	8	3	21	5	0.10
How to measurediabetes	155	8	123	11	<0.001	225	9	53	11	0.18	30	13	42	9	0.15
Diabetes causes eyedisease	272	13	197	18	<0.001	406	16	63	13	0.07	39	16	70	15	0.32
Diabetes due togenetics	343	17	208	19	0.81	491	19	60	12	<0.001	55	23	73	16	0.007
Diabetes can beprevented by regularexercise	968	48	591	55	<0.001	1319	51	240	48	0.19	144	60	178	39	<0.001
Diabetes can becontrolled byreducingcarbohydrate	981	48	584	54	0.001	1352	52	213	42	<0.001	153	64	179	39	<0.001
Diabetes can becontrolled byreducing sweet	1023	50	613	57	<0.001	1403	54	233	46	0.001	165	69	185	41	<0.001
Diabetes can beprevented by quittingsmoking	919	46	546	51	0.003	1303	50	162	32	<0.001	139	58	183	41	<0.001

	Education level									Socio-economic status									
	No(N = 1462)		Primary(N = 921)		Secondary(N = 495)		SSC orabove(N = 226)		P fortrend	Insufficientfunds allthe time(N = 423)		Insufficientfunds someof the time(N = 1077)			Balanced(N = 1320)		Sufficientfunds mostof the time(M = 268)		P for trend
	n	%	N	%	n	%	n	%		n	%		N	%	N	%	n	%	
Diabetes	1314	90	854	93	477	96	226	100	<0.001	352	84		993	92	1256	95	254	95	<0.001
Glucose tolerancetest (GTT)	32	2	34	4	24	5	32	14	<0.001	8	2		34	3	61	5	18	7	<0.001
How to measurediabetes	71	5	86	9	61	12	60	27	<0.001	24	6		97	9	100	8	55	21	<0.001
Diabetes causeseyedisease	129	9	142	15	113	23	85	38	<0.001	52	12		139	13	209	16	64	24	<0.001
Diabetes is due togenetics	145	10	180	20	134	27	92	41	<0.001	41	10		149	14	280	21	78	29	<0.001
Diabetes can beprevented byregular exercise	593	41	505	55	299	61	162	72	<0.001	122	29		514	48	755	57	157	59	<0.001
Diabetes can becontrolled byreducingcarbohydrate	607	42	504	55	289	58	165	73	<0.001	122	29		515	48	761	58	158	59	<0.001
Diabetes can becontrolled byreducing sweet	640	44	525	57	305	62	166	73	<0.001	138	33		529	49	804	61	155	58	<0.001
Diabetes can beprevented byquitting smoking	602	42	443	48	261	53	159	70	<0.001	139	33		451	42	708	54	158	59	<0.001

### Rasch Analysis and the association of socio-demographic factors with knowledge scores

Of the total participants, 54/3104 (1.7%) had heard of all the nine items relevant to knowledge and 129 (4.2%) were not aware of any of the items. The mean (95% CI) knowledge scores of the total sample was −1.603 (−1.723, −1.483) where 1173 (38%) participants had a knowledge score of zero or above i.e., equivalent to above average level of knowledge. After multivariable adjustment, males had significantly higher knowledge scores compared to females, β (95% CI), 0.393 (0.142, 0.631), p<0.001; every 10 years of older age was associated with lower knowledge score, −0.201 (−0.305, −0.098), p<0.001); and every incremental level of education was associated with higher knowledge score compared to no schooling, 0.726 (0.596, 0.857), p<0.001. People with sufficient funds most of the time had higher knowledge scores compared to those with insufficient funds (Table 3).

**Table 3:** Associations of Socio-demographic characteristics with the persons measures for all nine items.

	β (95% CI)*	p	β (95% CI)**	P
Gender:Male vs Female	0.563 (0.311, 0.814)	<0.001	0.393 (0.142, 0.643)	<0.001
Age per 10 years	−0.314 (−0.416, −0.211)	<0.001	−0.201 (−0.305, −0.098)	<0.001
Person with diabetes	0.719 (0.515, 0.923)	<0.001	0.583 (0.388, 0.778)	<0.001
Level of education (ref: no schooling)	0.980 (0.858, 1.102)	<0.001	0.726 (0.596, 0.857)	<0.001
SES (ref: always insufficient funds)	0.851 (0.710, 0.992)	<0.001	0.602 (0.462, 0.742)	<0.001
Hindu vs Mulim	−0.937 (−1.1261, −0.631)	<0.001	−0.816 (−1.123, −0.508)	<0.001

*Unadjusted β (95% confidence interval);

**adjusted for variables in the model.

The knowledge scores from all nine items combined were categorised into a binary cut-off of at greater or equal to zero (at least average level of knowledge) and less than zero (below average knowledge). The association of knowledge scores (i.e., the binary cut-off) with socio-demographic characteristics showed 41% male compared to 36% female (p = 0.004), 49% people with sufficient funds most or all of the time compared to 18% people with insufficient funds most of the time (p<0.001), and 68% people with at least SSC level of education compared to 29% with no schooling (p<0.001) had above average knowledge based on all nine items.

**Figure 2:**
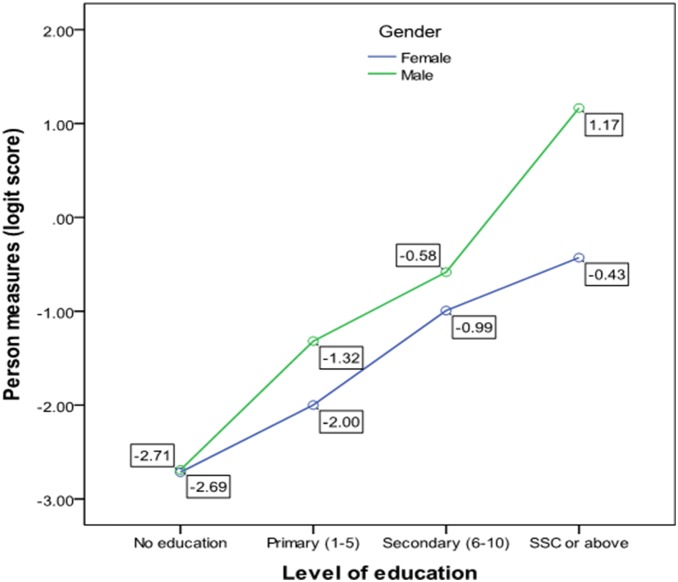
Interaction between gender and level of education (age adjusted) in total knowledge score.

There was a significant interaction effect for knowledge scores between gender and level of education (p = 0.02). Though knowledge scores were significantly higher for males, this difference was only apparent for those with at least SSC level of education (Figure 2).

### Attitudes towards treatment of diabetes

After adjusting for the covariates, individuals aged under 35 years were significantly more likely to report positive attitude towards the treatment for diabetes compared to those aged 65 years or more, odds ratio (OR) (95% CI) 1.73 (1.22, 2.43), p = 0.002. Gender, religion or educational attainment did not show any significant associations with attitude towards treatments for diabetes (Table 4).

**Table 4:** Associations of socio-demographic characteristics with attitudes (Should they need to seek treatment?) towards treatment of DM.

	No or do not know, 2034 (65.5%)		Yes, 1072 (34.5%)			
	n	%	n	%	OR (95% CI)*	P
Age, years						
Less than 35	134	56.3	104	43.7	1.73 (1.22, 2.43)	0.002
35–44	567	64.6	311	35.4	1.30 (1.01, 1.68)	0.05
45–54	620	65.8	322	34.2	1.21 (0.94, 1.55)	0.14
55–64	397	67.3	193	32.7	1.10 (0.85, 1.44)	0.47
Above or equal to 65	314	68.9	142	31.1	1.0	
Gender						
Female	1325	65.2	707	34.8	1.0	
Male	707	66.0	365	34.0	1.01 (0.86, 1.19)	0.89
Religion						
Muslim	1714	65.9	885	34.1	1.0	
Hindu	318	63.0	187	37.0	1.22 (1.0, 1.49)	0.05
Level of Education						
No education	955	65.3	507	34.7	1.0	
1–5 primary	614	66.7	307	33.3	0.87 (0.73, 1.05)	0.14
6–10 High school	332	67.1	163	32.9	0.79 (0.63, 1.0)	0.05
SSC or above	131	58.0	95	42.0	1.06 (0.78, 1.44)	0.73
Education						
Below SSC	1901	66.1	977	33.9	1.0	
SSC or above	131	58.0	95	42.0	1.18 (0.88, 1.59)	0.21
Socio-economic condition						
Insufficient funds all the times	303	71.6	120	28.4	1.0	
Insufficient funds some of the times	769	71.4	308	28.6	1.01 (0.78, 1.29)	0.96
Balance	792	60.0	528	40.0	1.67 (1.32, 2.14)	<0.001
Sufficient funds most of the time	156	58.2	112	41.8	1.88 (1.34, 2.63)	<0.001

*Odds Ratio (OR) (95% Confidence interval (CI)) adjusted for age, gender, education level, SES, diabetes status, and religion.

### Practice

The people with known diabetes (n = 99, 3%), 31 (31%) checked blood glucose levels once a year, 16 (16%) checked it at least twice in a year and the remaining had not had their blood sugar checked since diagnosis (Table 5). In relation to checking blood sugar levels, there was no significant difference across the socio-demographic parameters, with the exception of SES, where those with insufficient funds most or all of the time checked their blood glucose less frequently compared to those with sufficient funds most or all of the time (50% participants with sufficient fund most or all of the time checked blood glucose at least twice in a year compared to 6% participants with insufficient fund most of the time check blood glucose at least twice in a year, p = 0.001).

**Table 5:** Associations of socio-demographic characteristics with blood glucose check since diagnosis in people with know DM (n = 99).

	Did not check since diagnosis (n = 52, 53%)		One in a year (n = 31, 31%)		2–3 times in a year (n = 16, 16%)		
	n	%	n	%	n	%	P
Age, years							0.91
Less than 35	2	3.8	2	6.5	0	0	
35–44	8	15.4	13	41.9	2	12.5	
45–54	20	38.5	7	22.6	7	43.8	
55–64	13	25.0	4	12.9	3	18.8	
Above or equal to 65	9	17.3	5	16.1	4	25.0	
Gender							0.64
Female	32	61.5	16	51.6	10	62.5	
Male	20	38.5	15	48.4	6	37.5	
Level of education							0.15
No education	19	36.5	9	29.0	3	18.8	
Primary (1–5)	12	23.1	10	32.3	4	25.0	
Secondary (6–10)	14	26.9	4	12.9	5	31.3	
SSC or above	7	13.5	8	25.8	4	25.0	
Socio-economic condition							0.001
Insufficient funds all the times	12	23.1	2	6.5	1	6.3	
Insufficient funds some of the times	12	23.1	7	22.6	2	12.5	
Balance	22	42.3	16	51.6	5	31.3	
Sufficient funds most of the time	6	11.5	6	19.4	8	50.0	

## Discussion

This study shows that the level of knowledge associated with diabetes management and its risk factors is low in rural Bangladesh. We found that only 1173 (38%) participants had heard of a maximum of five knowledge items out of nine in regards to diabetes. Approximately half of our study population reported the knowledge that quitting smoking and controlling diet would benefit their diabetes management and diabetes would causes eye disease. Parameters important to self and clinical diabetes management, such as glucose tolerance test and standard glucose test were known to less than 5% and 10% in our study populations, respectively. Differences in positive attitudes towards the treatment of diabetes was largely explained by socio-economic status. In regards to the practice of diabetes, above 50% of the participants with known diabetes did not check their blood glucose level since diagnosis.

Previous studies both in developed and developing countries have reported that knowledge about diabetes is generally poor among diabetic patients [6], [23]–[26]. Similar to our findings, one study that surveyed patients attending tertiary education hospitals in Gujrat-the Saurashtra region of India [26] found that 51% of patients believed exercise assisted with diabetes control, 75% knew that diet was important in diabetes control and only 7% reported quitting smoking was related to diabetes management. Another study of 575 patients with diabetes in the United Arab Emirates attending outpatient clinics [6] reported that 60% of people believed that diabetes was caused by excessive sugar and sweets. This concurs with our findings that 53% of participants believed diabetes could be controlled by reducing sweet and sugar.

In 2012, Saleh et al. [13] conducted a KAP survey in people with diabetes enrolled in the health care centres in urban cities in Bangladesh and found that 82% people had at most an average level of basic knowledge of diabetes computed by using a modified version of the Diabetes Knowledge Test (DKT), a questionnaire validated by the University of Michigan [27]. However, our Rasch analysis showed that overall the participants have a significantly below average knowledge score, and 62% had at most an average level of knowledge. Most of the previous studies including the study conducted in Bangladesh examined those who were already diagnosed with diabetes and attending hospitals or centres for diabetes care, and thus the responses may be biased compared with our findings from the general population. If prevention is to be effective therefore, diabetes education needs to reach those who are not enrolled in, or engaged with a health care centre.

With regard to the risk factors of explaining the variance of poor knowledge, attitudes and practice, Al-Maskari et al [6] reported gender, age and SES effects on knowledge. For instance, higher total knowledge scores were found for males (males 17.08 vs females 15.26, P<0.001) and people with a post-graduate education (post-graduate education 19.67 compared to those with less than post-graduate education 14.74, P<0.001). However, the authors did not find any significant difference in attitudes towards the ability to self-manage diabetes between gender, age groups, and income levels. Both knowledge score and attitudes towards treatments for diabetes in our population are very similar to those reported by Al-Maskari et al [6]. However, our study reports that the people with insufficient funds had significantly less positive attitudes towards treatments or intention of treatment of diabetes which can be attributed to the difference in socio- economic factors between UAE and Bangladesh. For example, the per capita gross domestic product (GDP) in UAE in 2012 was US$48158.00 compared to $848.00 in Bangladesh. Therefore, we speculate that family income is a major contributory factor in changing attitudes towards treatment of any disease unless the patients are in a critical need to go to the emergency treatment in low income countries like Bangladesh.

The significantly higher level of knowledge in males, younger age groups, higher education attainment and better socio-economic status were primarily explained by level of education. The literacy level in females is lower than that of their male counterparts, and the level of education is higher in the younger generation than the older generation [28] which are also evident from our current study. Though a significant difference in knowledge scores was observed between males and females, with the changes in social trends and the increase in female uptake in primary and secondary education [28] this difference may diminish in coming years. Significant differences in knowledge scores between Hindu and Muslim may also be attributed to the lower education level amongst lower minority casts in the Hindu religion such as in Cobbler and Fishermen. Given that these religions were dominant within the region from which participants in this study were recruited, the results relating to knowledge and religion may not be generalised at the national level.

In developing countries, many misconceptions remain about the nutritional advice for people with diabetes. The myth still remains that the reduction of sugar and carbohydrate controls diabetes. Rather, the main aim is to reduce total weight through lifestyle behavioural practice including increasing the amount of exercise, reducing the intake of highly refined foods, including more legumes, vegetables, whole grains and fruits, and reducing or stopping smoking [29], [30]. This research hints at the possibility that informal sources of health promotion information cannot be relied upon in Bangladesh, and greater efforts need to be directed toward improving the understanding of health professionals and the community in general about the symptomatology and progression of the disease [2], [6], [29].

Our study provided the first reliable data on the KAP of diabetes and its risk factors not only in a rural community but also among the general population of Bangladesh. The analysis was based on a large data set of adults, from whom all relevant data were collected directly through face-to-face interview. The sophisticated Rasch analysis technique was applied to quantify the item responses in logarithmic scale from a binary scale that suffers from identifying the real difference between two binary outcomes.

The potential drawback of our study is a report from a single-occasion collection of data from a single location. Whilst we have attempted to capture the situation in Banshgram, the study would obviously need to be repeated in a random sample of other remote areas in order for the results to be truly representative of a national perspective.

In conclusion, health interventions and education programs must be appropriately planned and implemented at a national level to manage risk factors for diabetes, such as sedentary life style, dietary modification and conducting regular screening program to identify people with diabetes and pre-diabetes. This is imperative to improve the general level of knowledge on the self-management of diabetes to prevent the development of diabetic complications. The findings of the research suggests that public health interventions related to diabetes and its risk factors should target certain groups of the population particularly women and those with lower education and incomes to ensure benefits from individual, societal and health-economic perspectives.

## Supporting Information

Data S1 **BPDES data.**
(SAV)
